# Supplementary material for: Neural mechanisms underlying the hierarchical construction of perceived aesthetic value
Source: Nat Commun. 2023 Jan 24;14:127. doi: 10.1038/s41467-022-35654-y (PMC9873760; doi:10.1038/s41467-022-35654-y)
Supplement: Supplementary file 1 — Supplementary Information [file 41467_2022_35654_MOESM1_ESM.pdf]

Supplementary materials for “Neural mechanisms underlying the hierarchical construction of perceived aesthetic value”

Kiyohito Iigaya [1,2,3,+], Sanghyun Yi[1], Iman A. Wahle[1], Sandy Tanwisuth[1], Logan Cross[1,4], and John P. O’Doherty[1,*]

[1]Division of Humanities and Social Sciences, California Institute of Technology, 1200 E California Blvd, Pasadena, CA 91125
[2]Department of Psychiatry, Columbia University Irving Medical Center, New York, NY 10032
[3]Center for Theoretical Neuroscience and Mortimer B. Zuckerman Mind Brain Behavior Institute, Columbia University, New York, NY 10027, USA
[4]Department of Computer Science, Stanford University, Stanford, CA, USA.
[+]ki2151@columbia.edu
[*]jdoherty@caltech.edu

Supplementary figures

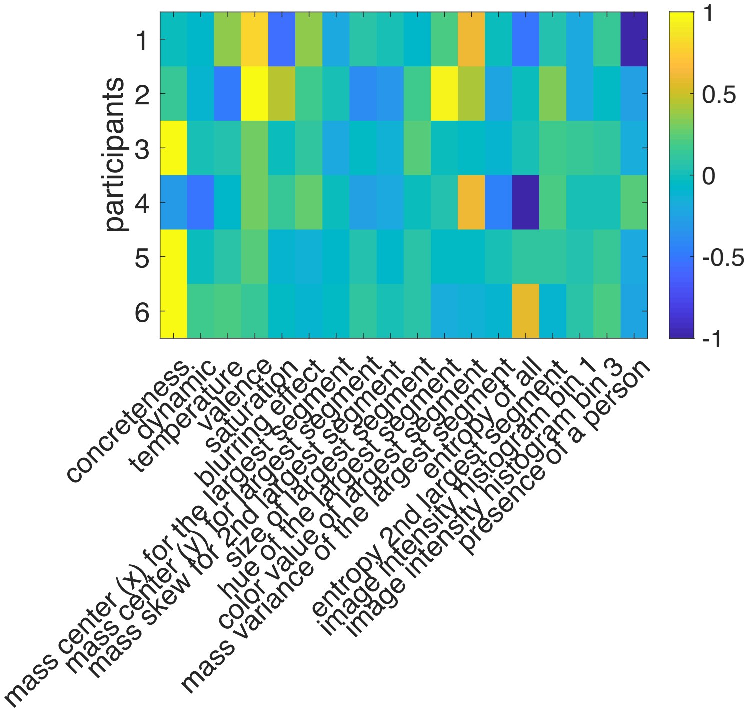

Supplementary Figure 1: Feature weights from each fMRI participant, determined by fitting the LFS model to the liking ratings from each participant.

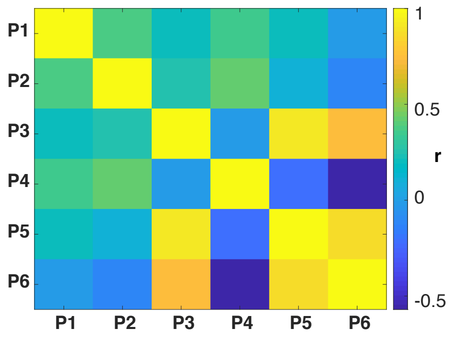

Supplementary Figure 2: Correlations between feature weights across fMRI participants.

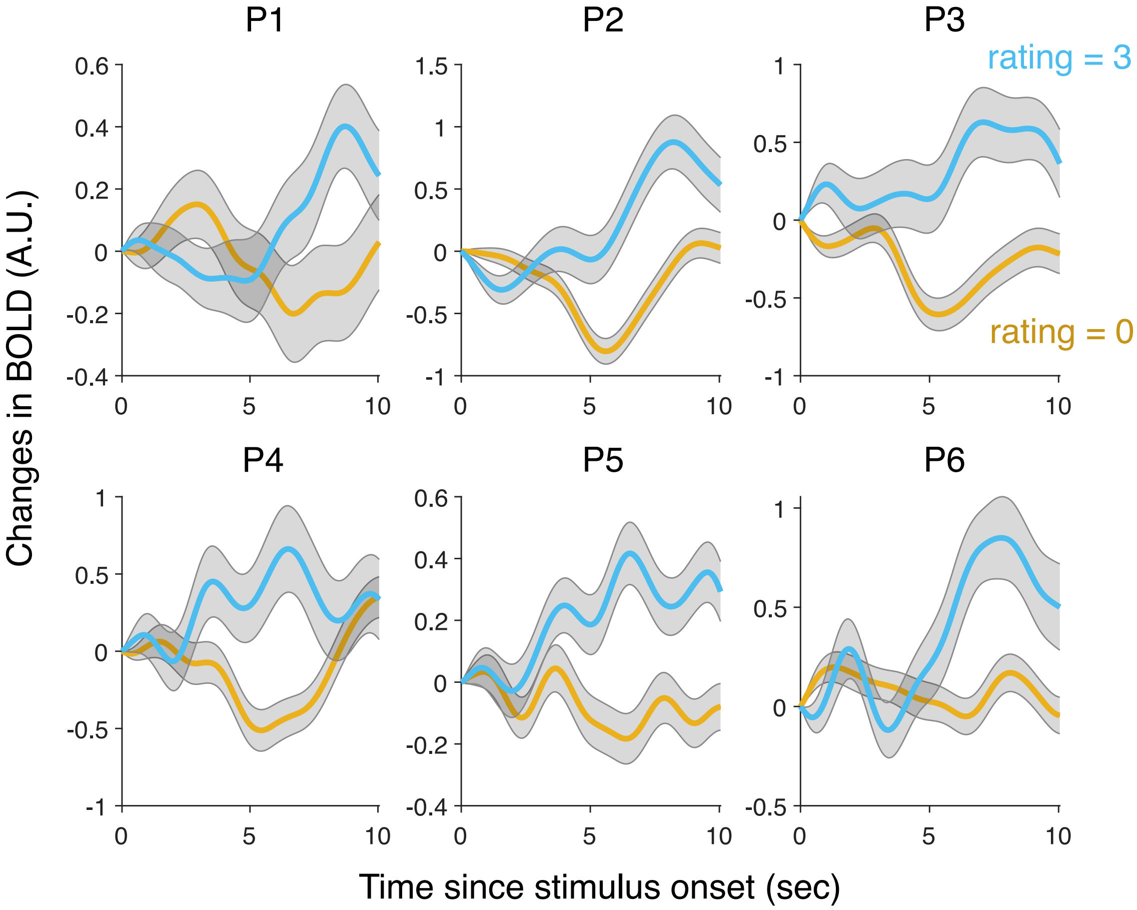

Supplementary Figure 3: The time course of BOLD signals in mPFC. BOLD signals are extracted from the cluster in mPFC correlating with liking ratings from each participant depicted in **Figure 3** in the main paper. The signals are up-sampled[?] and shown for trials in which participants gave the highest (liking rating =3; blue) and the lowest (liking rating =0; orange). The error bars indicate the mean ± SEM over trials.

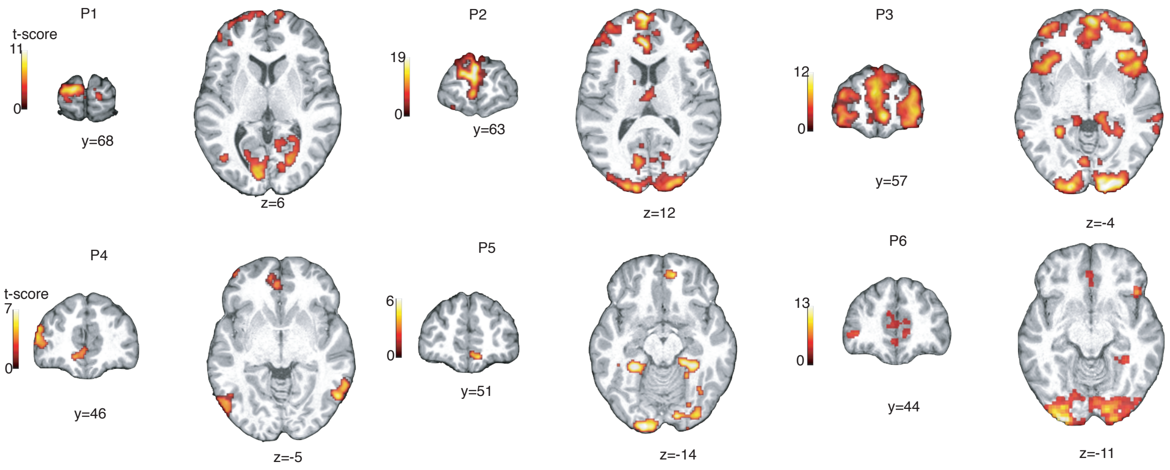

Supplementary Figure 4: Neural correlates of subjective value. One-sided t-test. An adjustment was made for multiple comparison correction: clusters at whole-brain cFWE $p < 0.05$ with height threshold at $p < 0.001$ are shown.

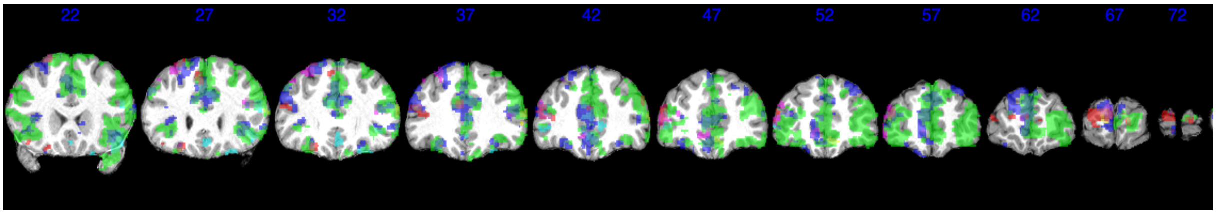

Supplementary Figure 5: Neural correlates of subjective value. One-sided t-test. An adjustment was made for multiple comparisons: clusters at whole-brain cFWE $p < 0.05$ with height threshold at $p < 0.001$ are shown for slices ranging from $y = 22$ and $y = 72$ for participant 1 (red), participant 2 (blue), participant 3 (green), participant 4 (violet), participant 5 (yellow), participant 6 (cyan).

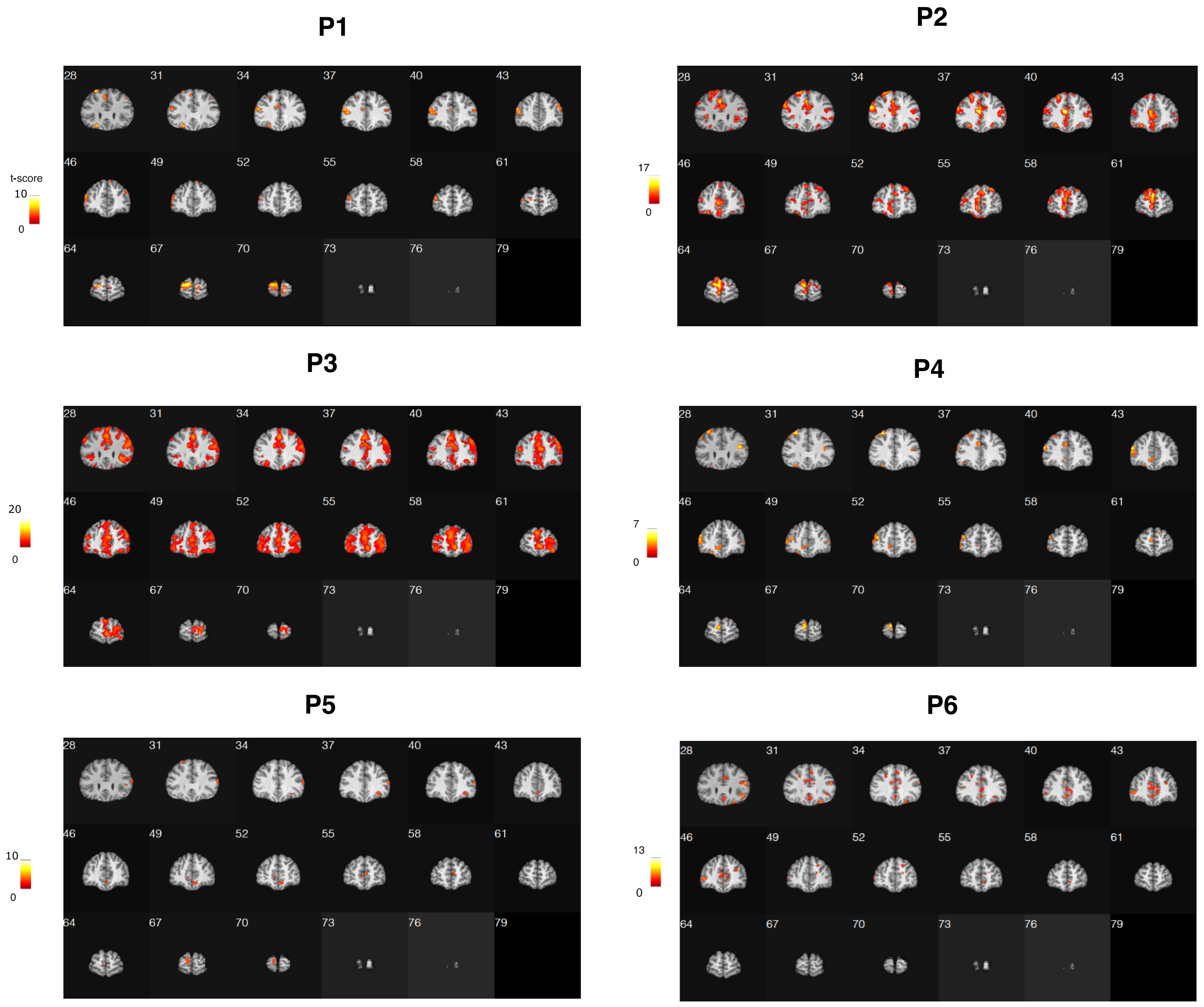

Supplementary Figure 6: Neural correlates of subjective value. Voxels at $p < 0.001$ uncorrected are shown for slices between $y = 28$ and $y = 79$. One sided t-test.

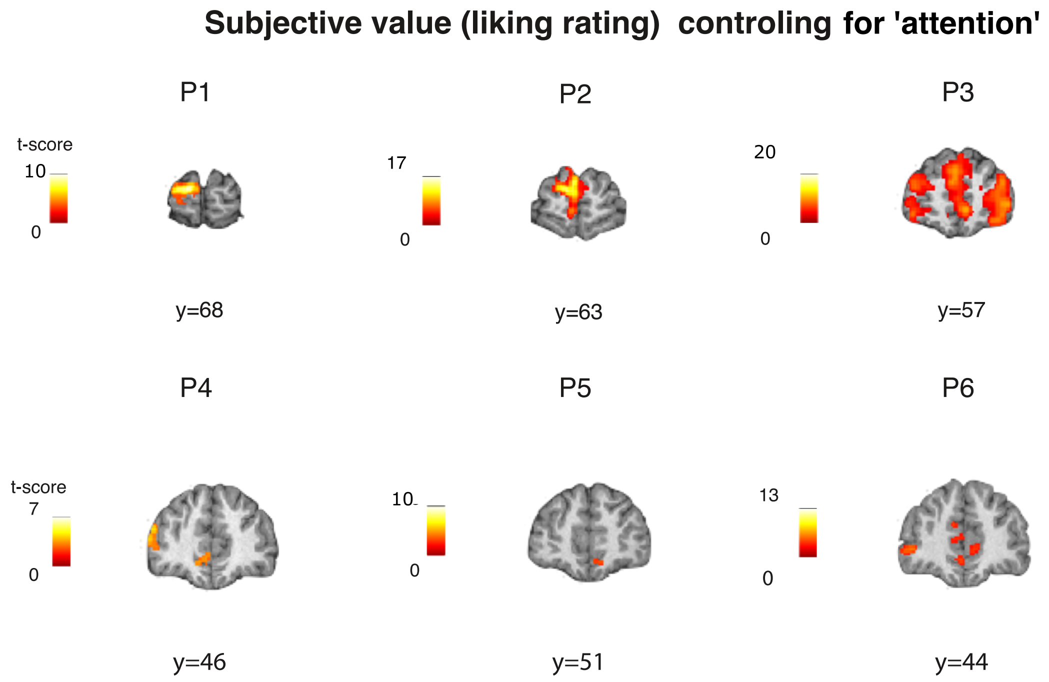

Supplementary Figure 7: Neural correlates of subjective value when controlling for the effects of attention (reaction time, squared reaction time, distance from the mean rating). One-sided t-test. An adjustment was made for multiple comparisons: clusters significant at whole-brain cFWE $p < 0.05$ with height threshold at $p < 0.001$ are shown.

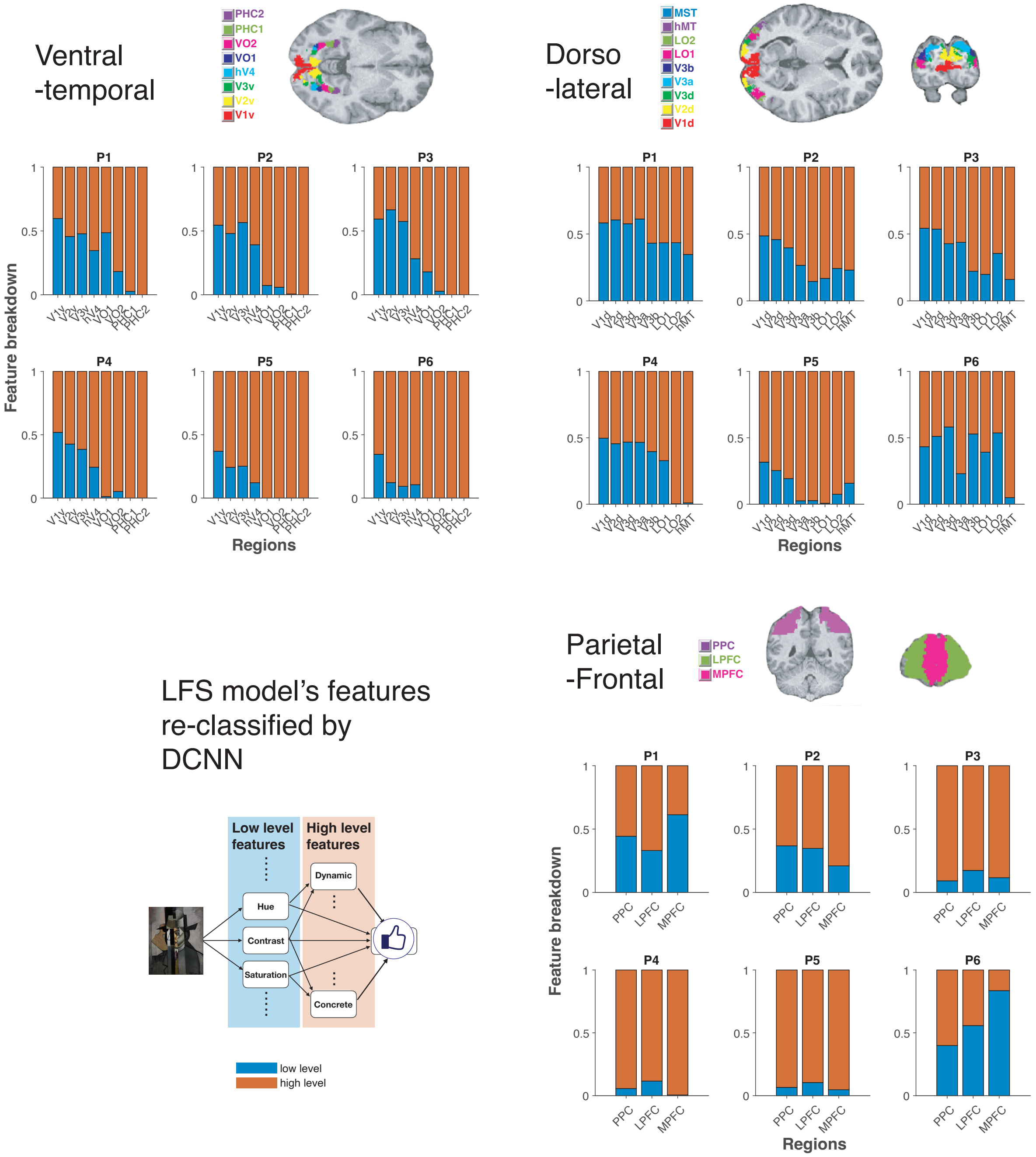

Supplementary Figure 8: Results of fMRI encoding analysis of low- and high-level features, using the features that are reclassified according to the DCNN results. Among the features that were originally considered, the features showing significantly positive slopes across layers in the DCNN were defined as high-level features, while the features showing significantly negative slopes across layers in the DCNN were defined as low-level features. The results did not qualitatively change from our original analysis with the original definition of low- and high- level features. Credit. Jean Metzinger, Portrait of Albert Gleizes (public domain).

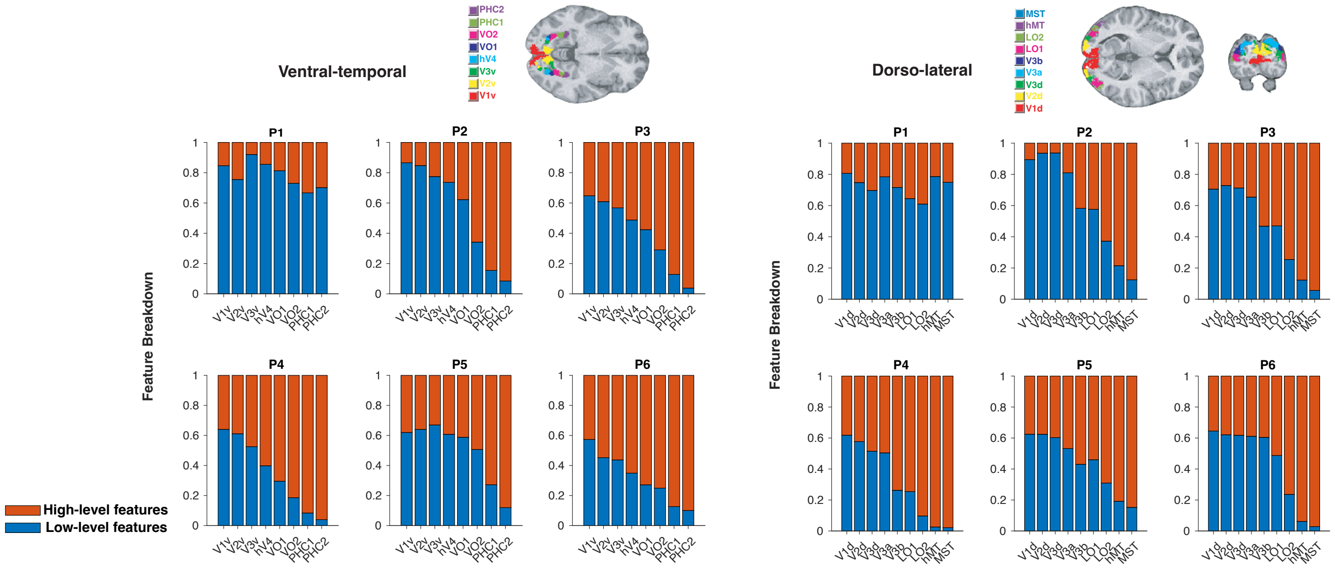

Supplementary Figure 9: Encoding analysis of low-level, high-level, features using lasso regression with cross validation within subject. The results of ROIs in the ventral-temporal visual stream are shown.

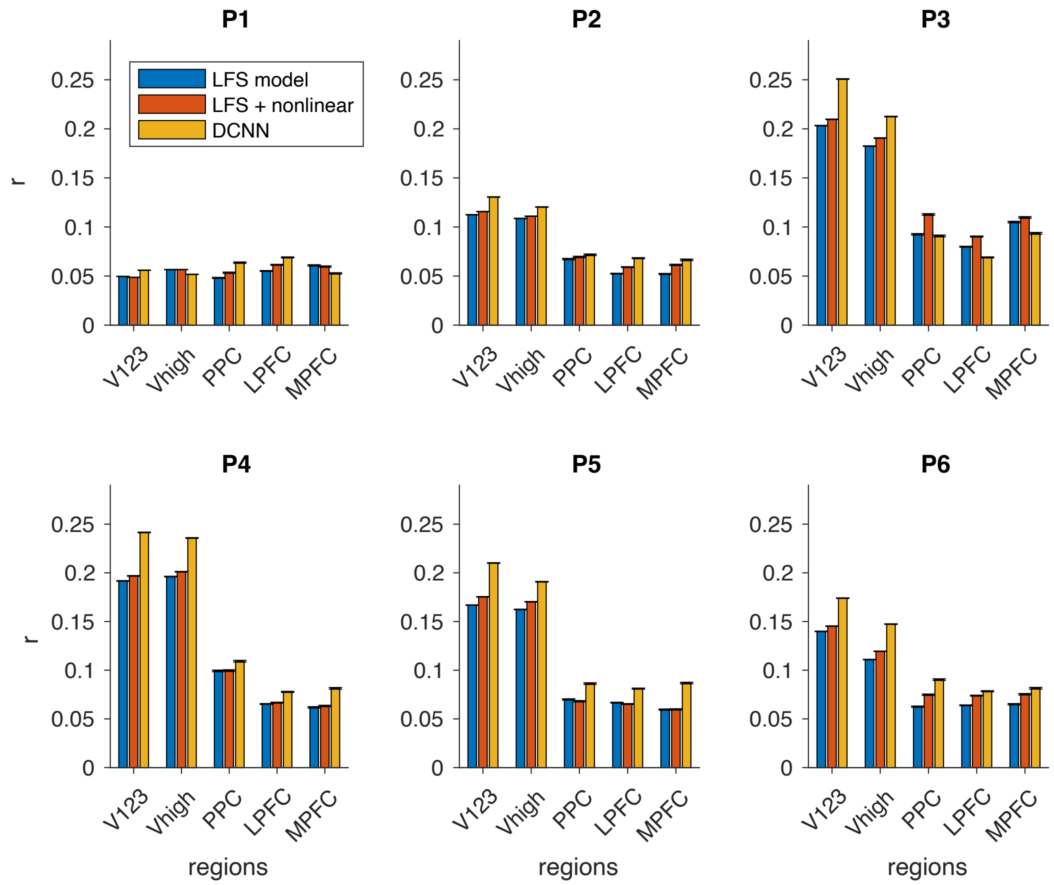

Supplementary Figure 10: Contrasting different model predictive accuracies, measured by Pearson correlations, across ROIs. Blue is the model with original low and high level features. Red is the model with original features and nonlinear features that are constructed by interactions between pairs of original features. Yellow is the DCNN hidden layers (150 PCs in total). The correlation was computed for each voxel for each participant. The colored bars indicate the mean and the error bars indicate standard errors across voxels in each ROI.The number of voxels varied across ROIs.

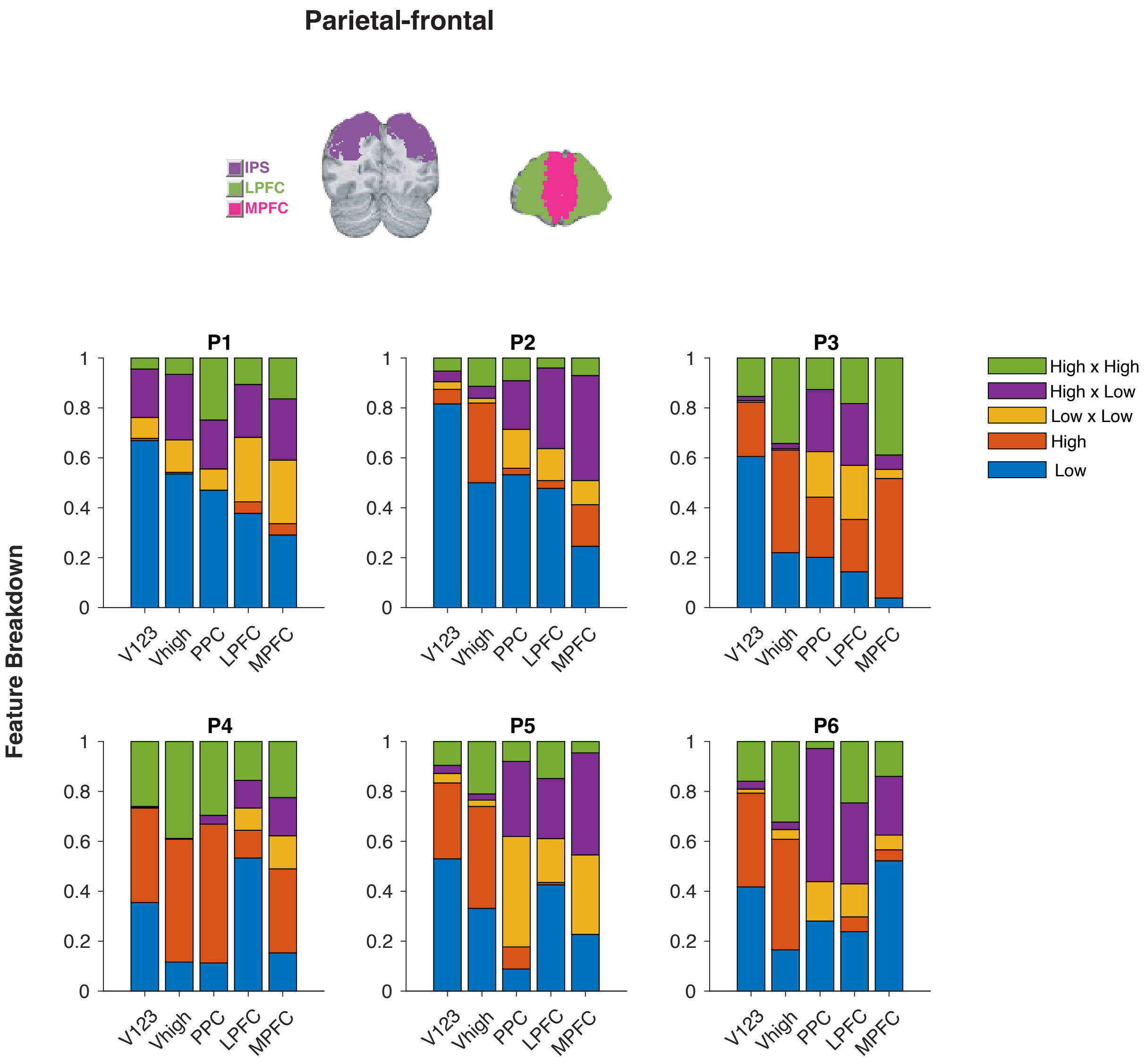

Supplementary Figure 11: Encoding analysis of low-level, high-level, and interaction term features (low x low, high x high, low x high), using lasso regression with cross validation within subject. The results of ROIs in visual areas, PPC, PFC are shown.

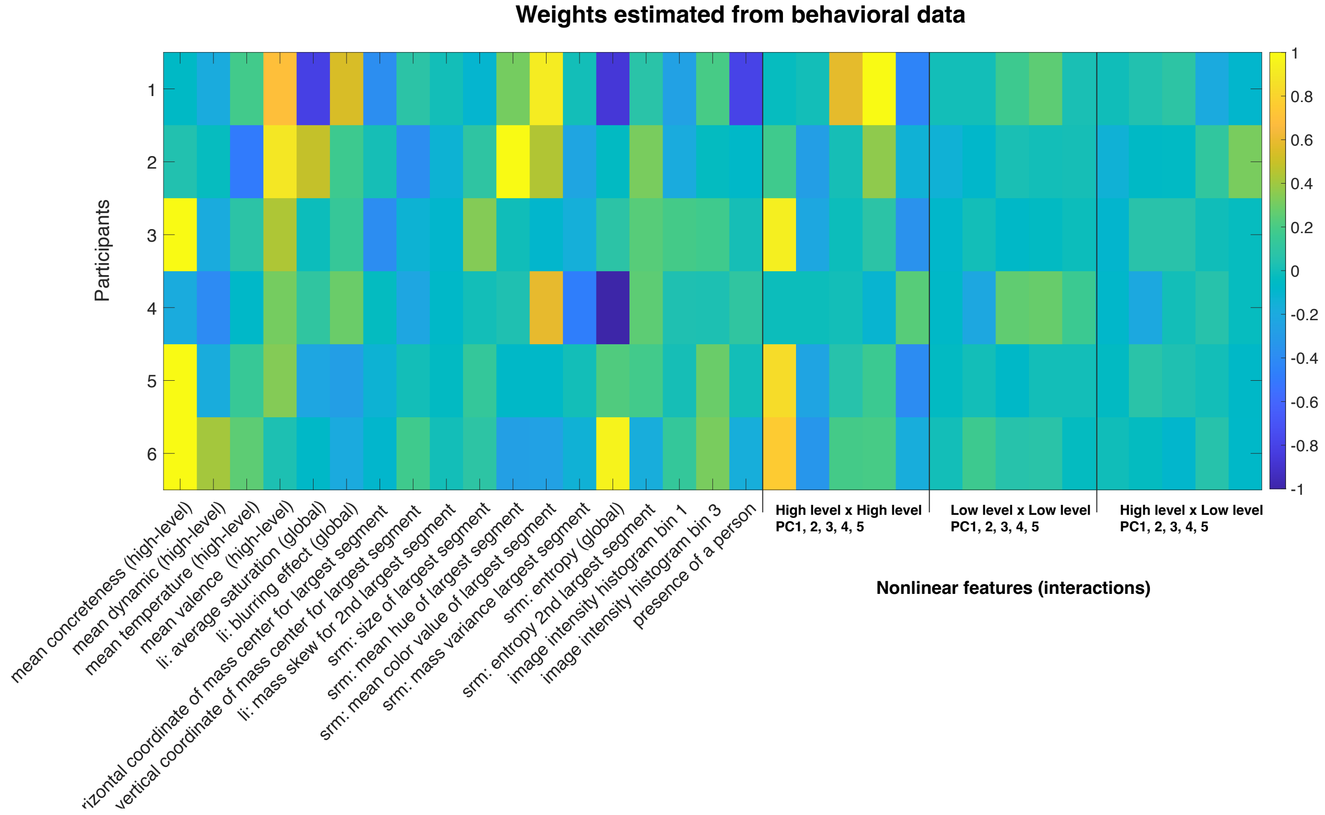

Supplementary Figure 12: The weights estimation of original and nonlinear features across partici-apnts.

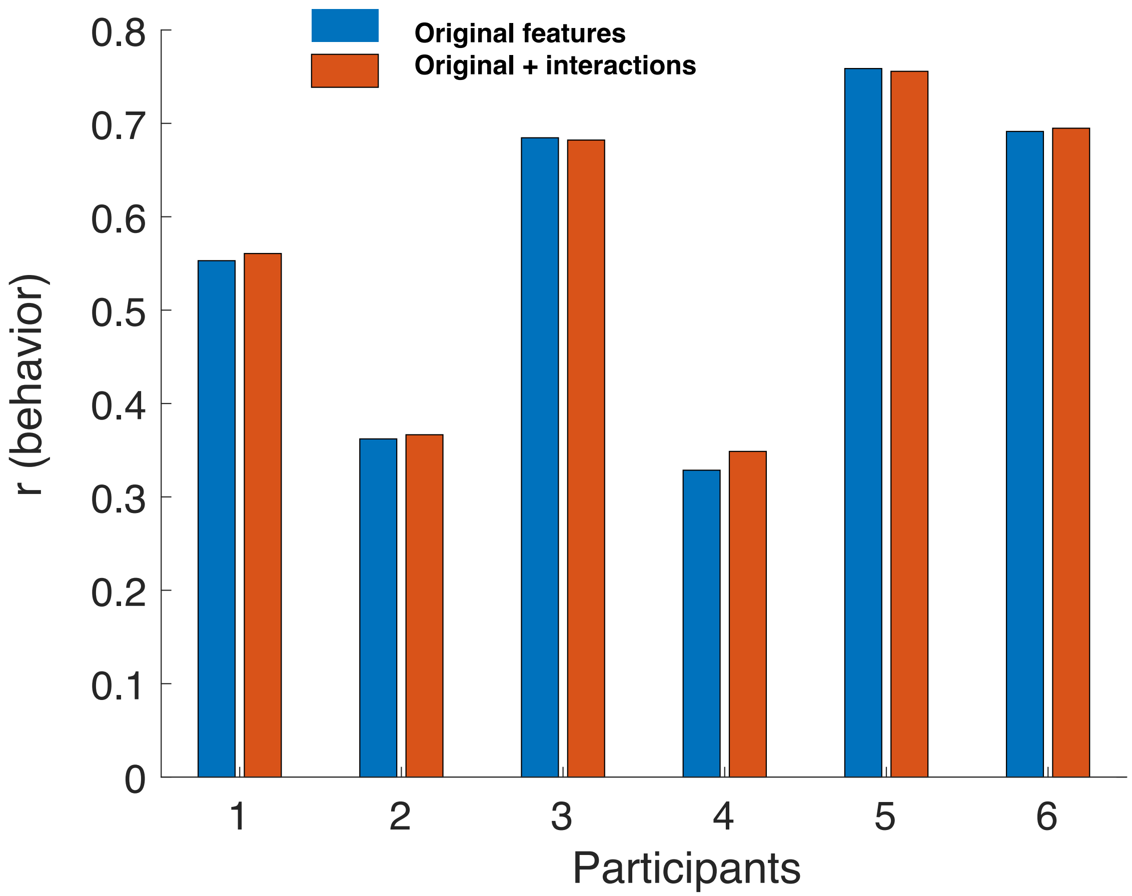

Supplementary Figure 13: Behavioral prediction with original low and high level features (blue), and with these features plus nonlinear interaction features (red)

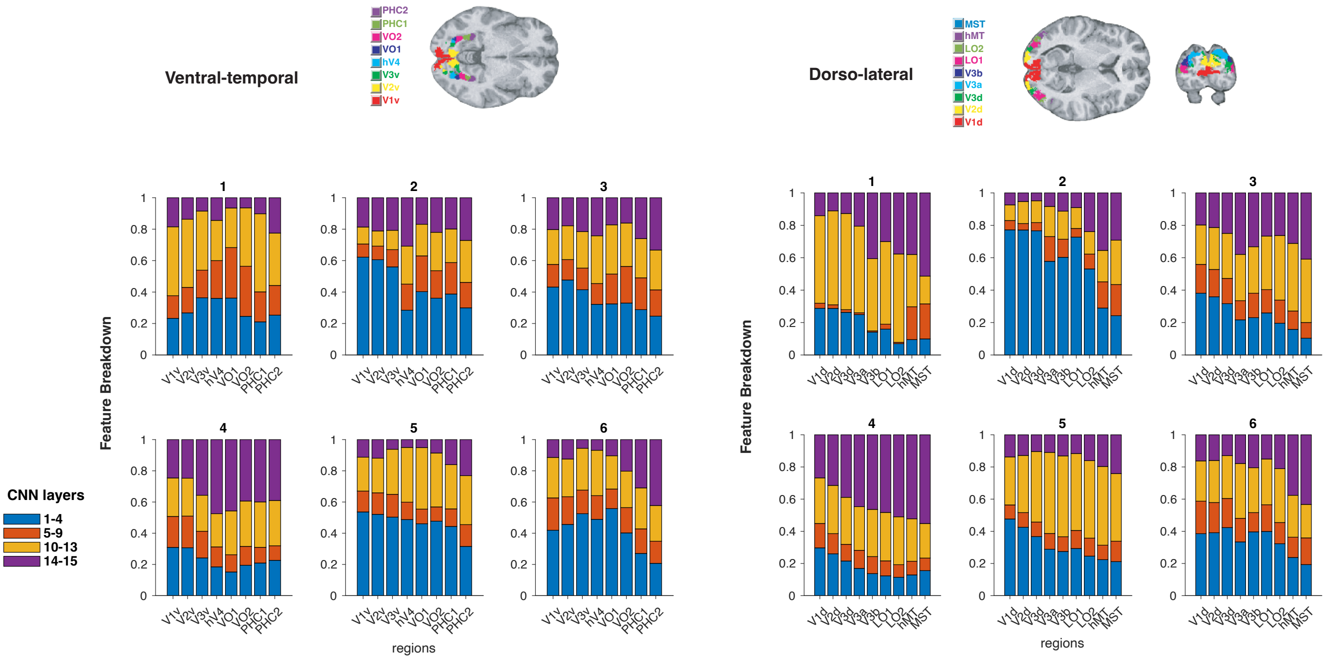

Supplementary Figure 14: Encoding analysis of DCNN features (45 features in total, 3 features per layer) using lasso regression with cross validation within subject. The results of ROIs in the ventral-temporal and dorso-lateral visual stream are shown.

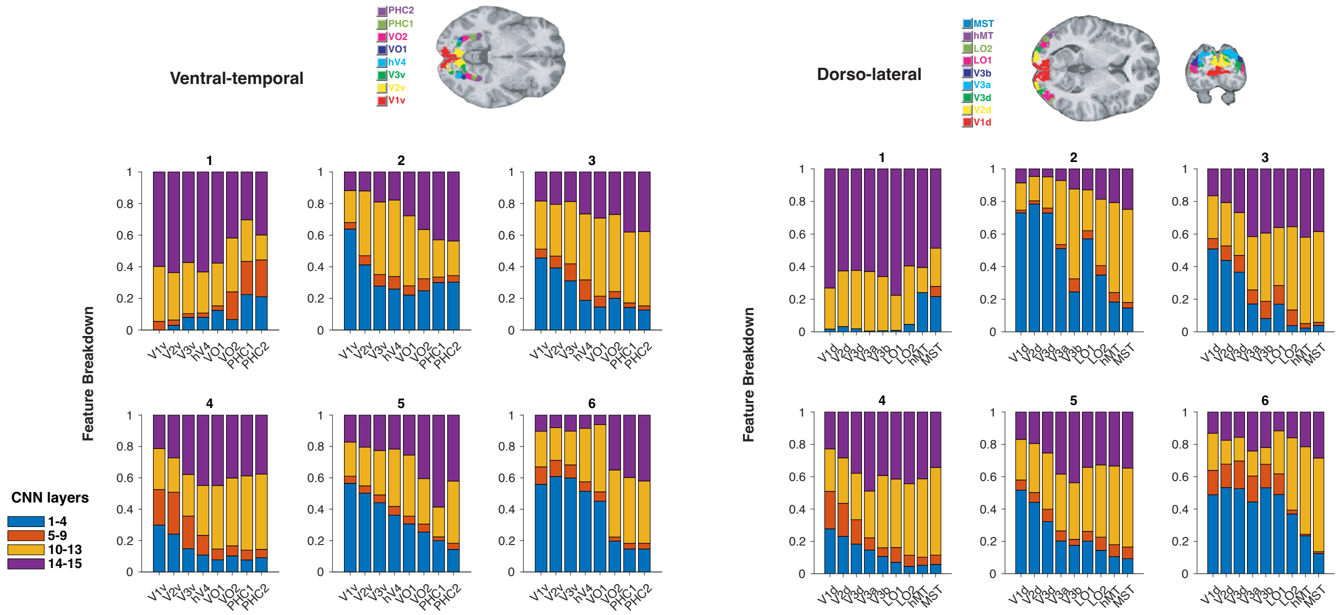

Supplementary Figure 15: Encoding analysis of DCNN features (150 features in total, 10 features per layer) using lasso regression with cross validation within subject. The results of ROIs in the ventral-temporal and dorso-lateral visual stream are shown.

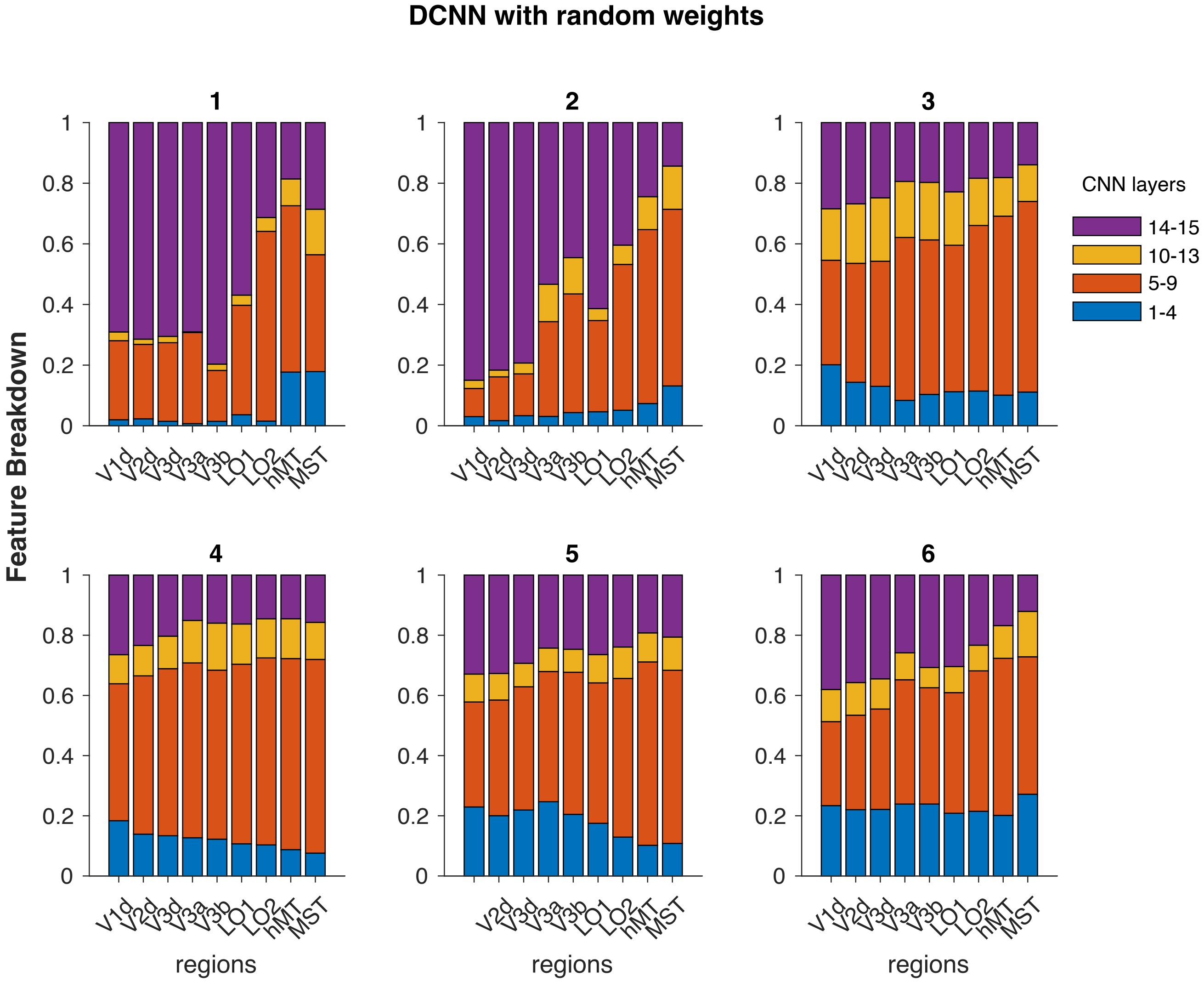

Supplementary Figure 16: Encoding analysis of the DCNN model features, where the model weights were set to random, using lasso regression with cross validation within subject. The results of ROIs in the ventral-temporal visual stream are shown.

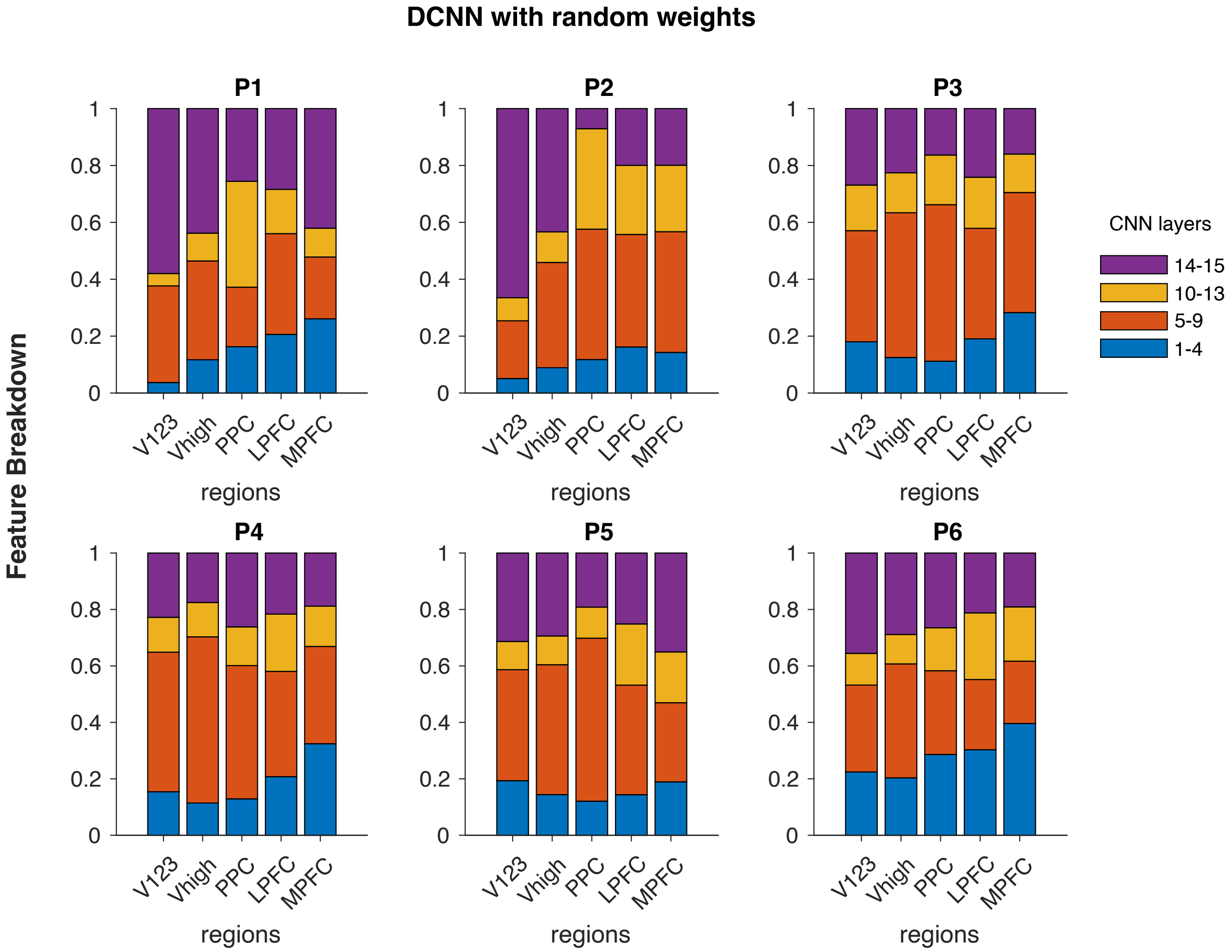

Supplementary Figure 17: Encoding analysis of DCNN model features, where the model weights were set to random, with lasso regression with cross validation within subject. The results of ROIs in visual areas, PPC, PFC are shown.

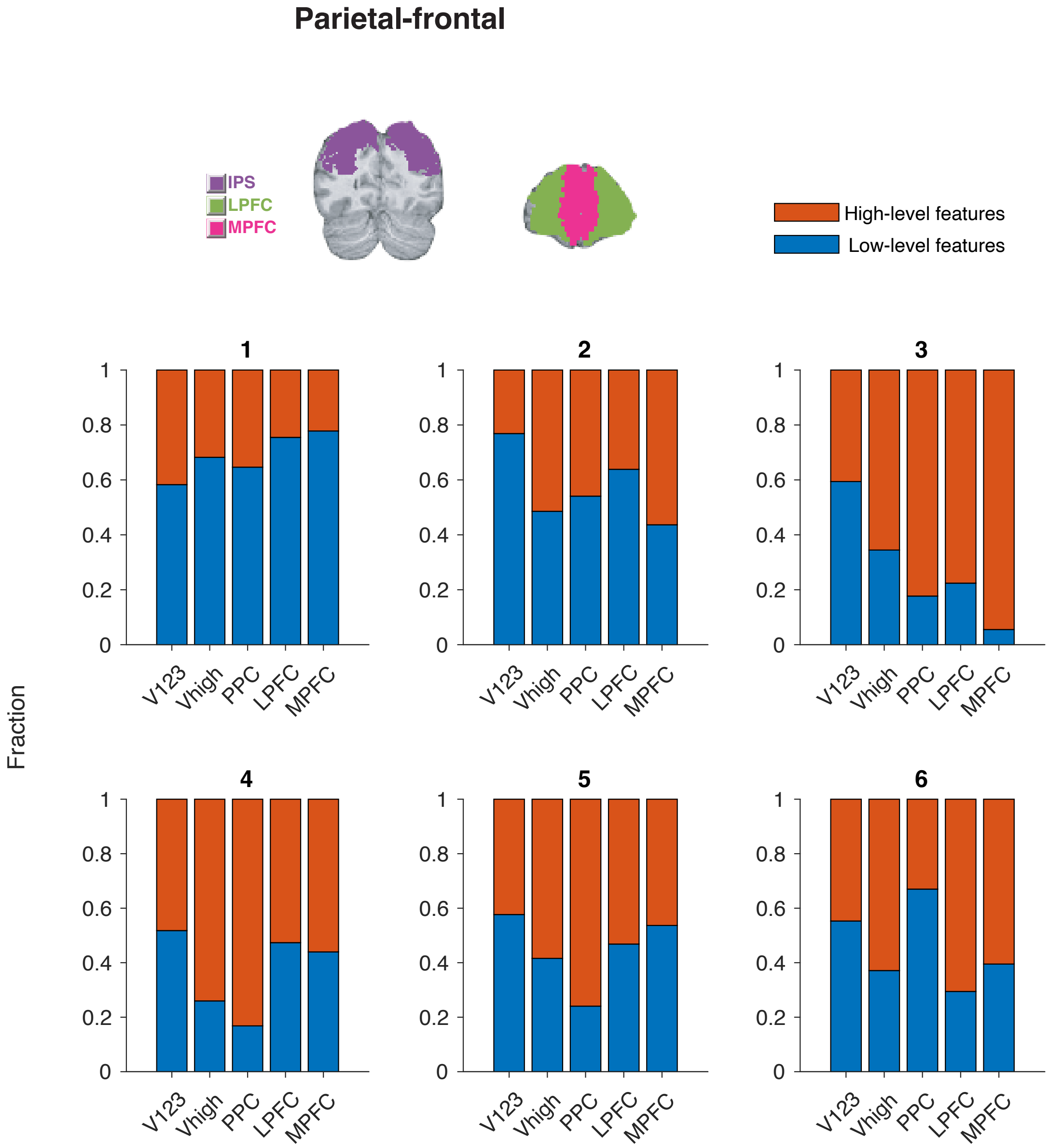

Supplementary Figure 18: Encoding analysis of low-level, high-level, features using lasso regression with cross validation within subject. The results of ROIs in visual areas, PPC and PFC are shown.

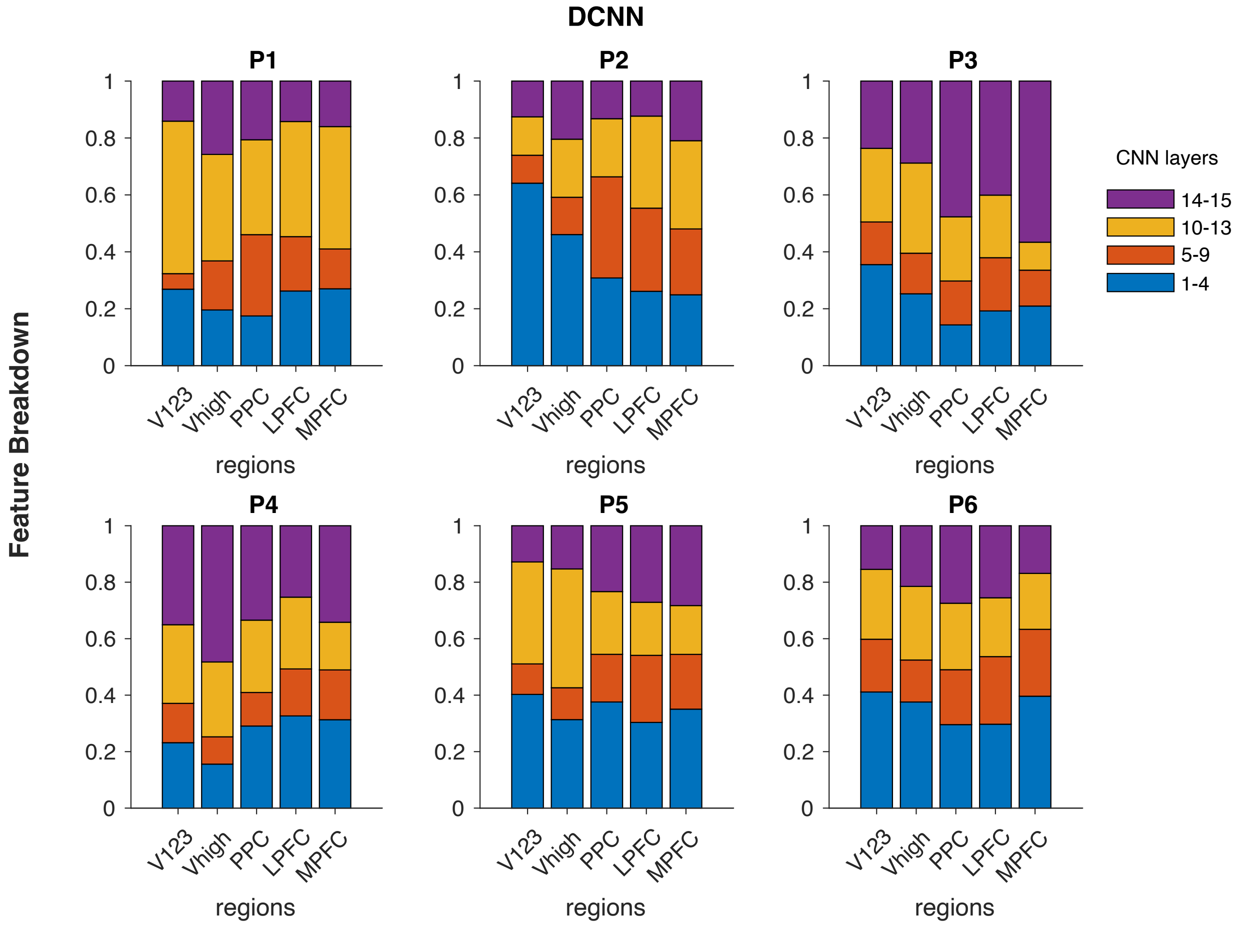

Supplementary Figure 19: Encoding analysis of DCNN features using lasso regression (45 features in total, 3 features per layer) using cross validation within subject. The results of ROIs in visual areas, PPC, PFC are shown.

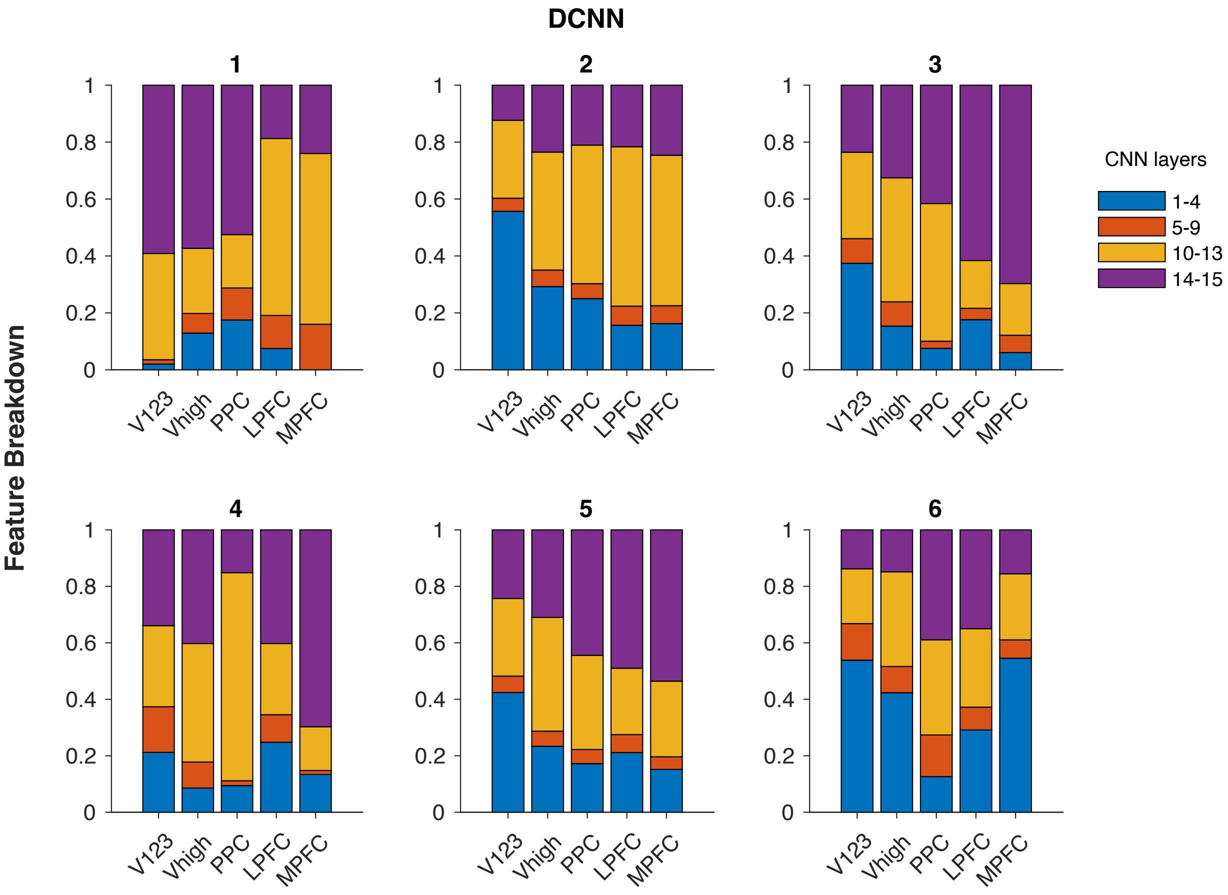

Supplementary Figure 20: Encoding analysis of DCNN features (150 features in total, 10 features per layer) using lasso regression using cross validation within subject. The results of ROIs in visual areas, PPC, PFC are shown.

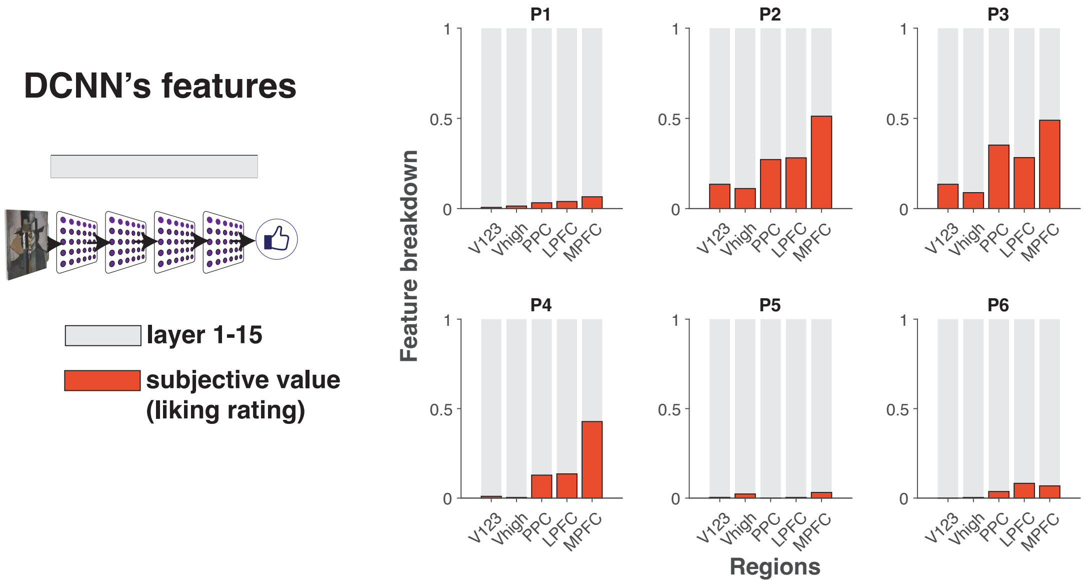

Supplementary Figure 21: The same analysis as Figure 7A but now with the DCNN model features. Credit. Jean Metzinger, Portrait of Albert Gleizes (public domain).

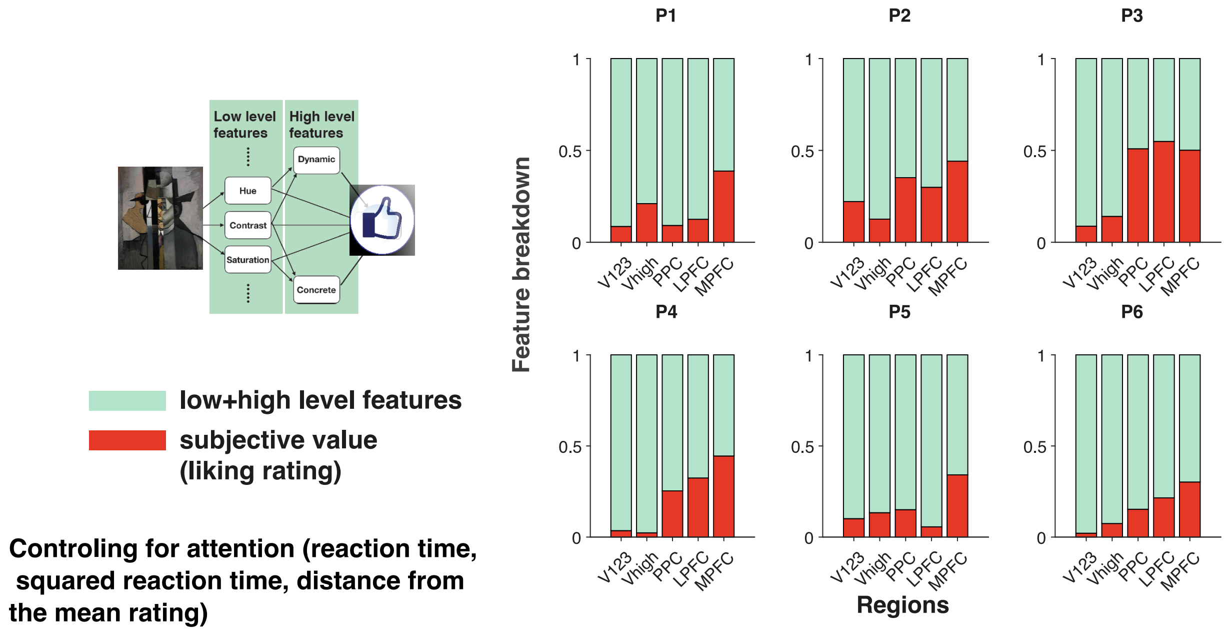

Supplementary Figure 22: The breakdown of feature representations and value representations across cortical regions when controlling for reaction time, squared reaction time, and distance from the mean rating. Credit.Jean Metzinger, Portrait of Albert Gleizes (public domain).

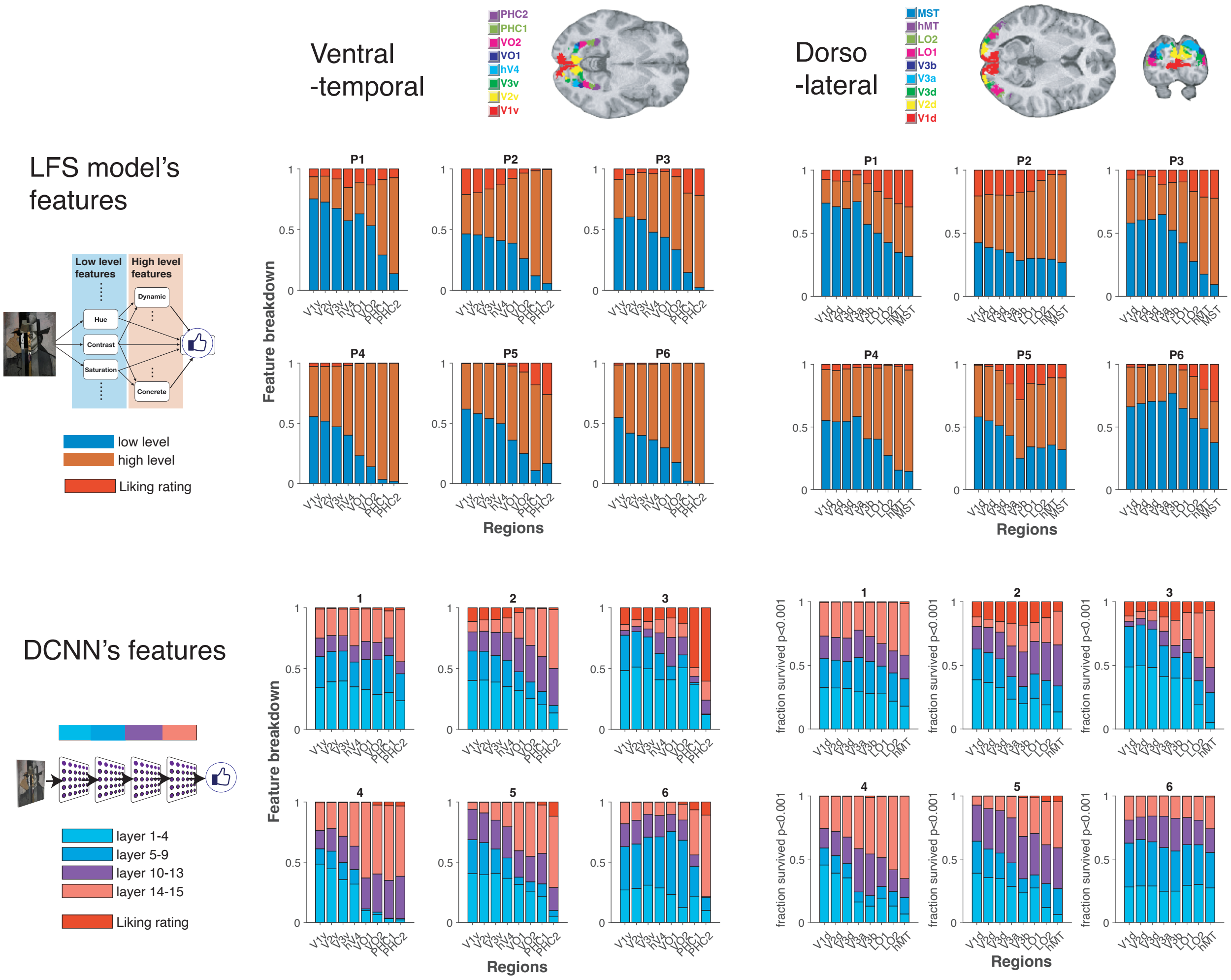

Supplementary Figure 23: Encoding analysis of low- and high-level features when subjective liking ratings are also included into the same GLM. The results of ROIs in the ventral-temporal and dorso-lateral visual streams are shown. Credit. Jean Metzinger, Portrait of Albert Gleizes (public domain).

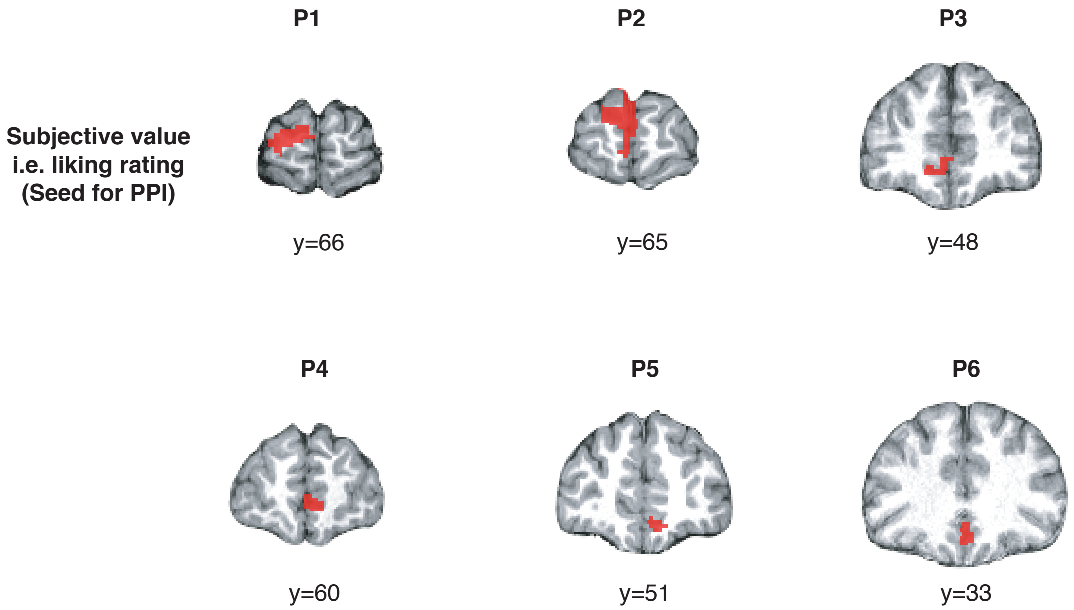

Supplementary Figure 24: The seeds of the PPI analysis. The seeds used for the PPI analysis correspond to a medial PFC cluster drawn from each participant that was found to show significant correlation with subjective value. The cluster used for each participant is shown here (One-sided t-test. An adjustment was made for multiple comparisons: $p < 0.05$ cFWE at whole-brain with a height threshold of $p < 0.001$).

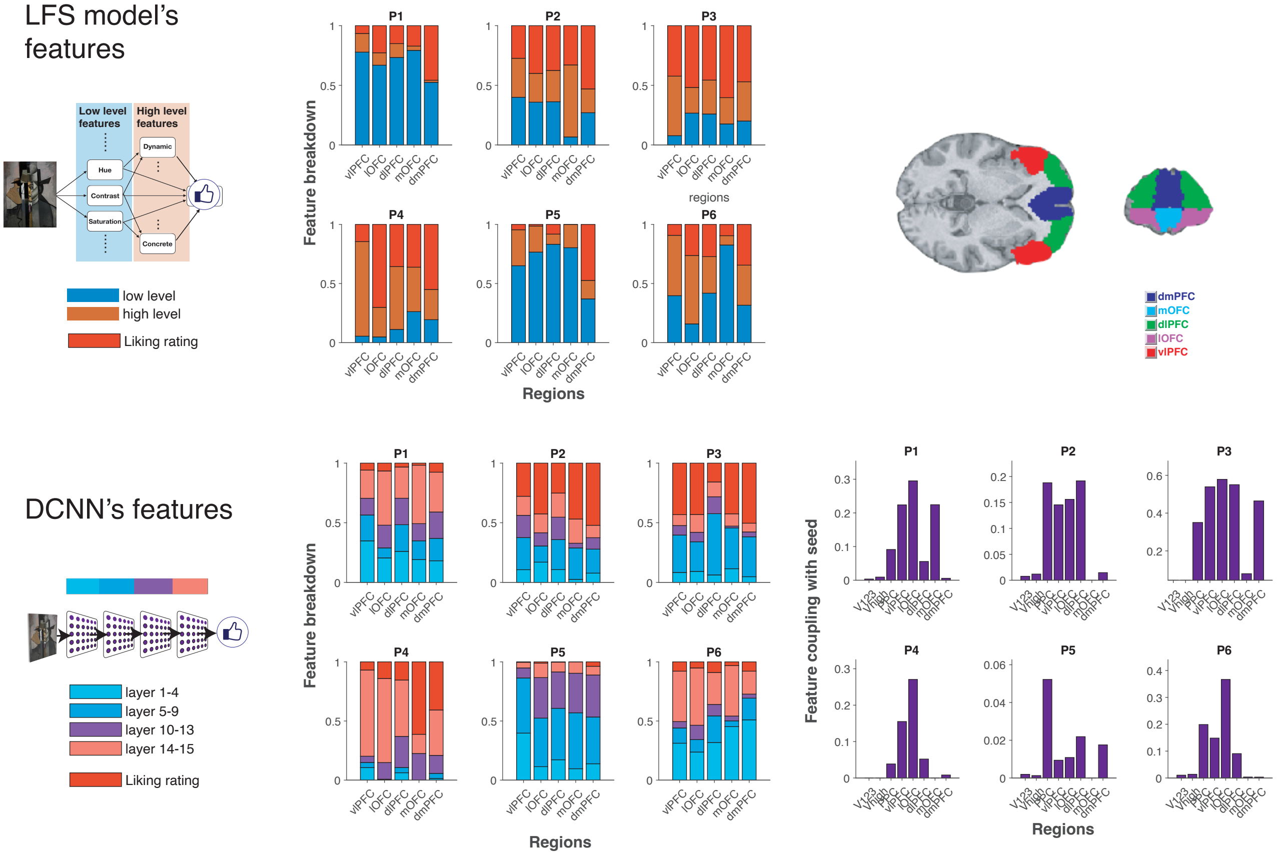

Supplementary Figure 25: The same results as in Figures 7 and Supplementary figure 21, but now broken down to show separate results for each sub-region of the PFC. Credit. Jean Metzinger, Portrait of Albert Gleizes (public domain).

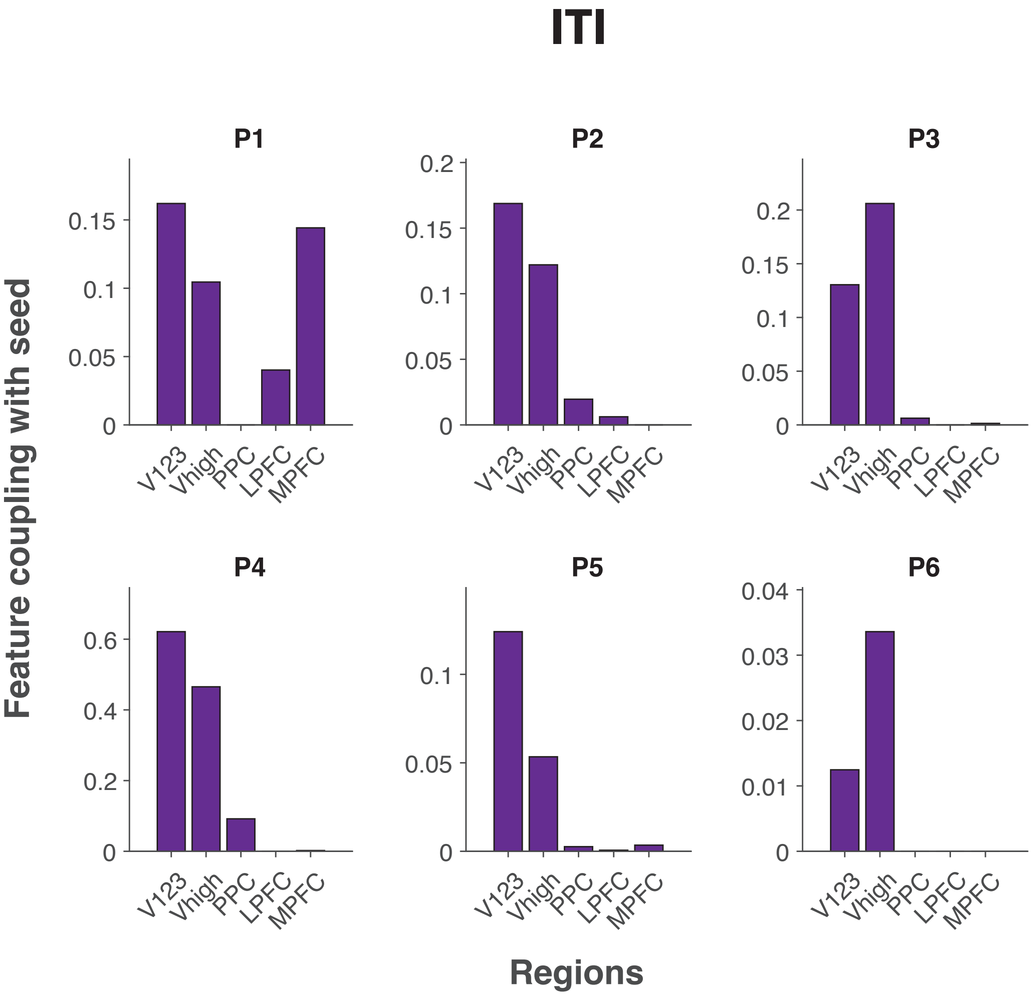

Supplementary Figure 26: The same analysis as in Figure 7C, except here the epoch of the ITIs are taken as the psychological regressor, as opposed to the epoch of presentation of the visual stimuli. In this situation, we did not observe robust coupling between mPFC value areas and lateral PFC and PPC, thereby supporting the possibility that increased coupling between lPFC, PPC and mPFC occurs specifically at the time of stimulus evaluation.
