# Supplementary material for: Neural mechanisms underlying the hierarchical construction of perceived aesthetic value
Source: Nat Commun. 2023 Jan 24;14:127. doi: 10.1038/s41467-022-35654-y (PMC9873760; doi:10.1038/s41467-022-35654-y)
Supplement: Supplementary file 3 — Reporting Summary [file 41467_2022_35654_MOESM3_ESM.pdf]

Corresponding author(s): Kiyohito Iigaya

Last updated by author(s): Nov 10, 2022

Reporting Summary

Nature Research wishes to improve the reproducibility of the work that we publish. This form provides structure for consistency and transparency in reporting. For further information on Nature Research policies, see our Editorial Policies and the Editorial Policy Checklist.

Statistics

For all statistical analyses, confirm that the following items are present in the figure legend, table legend, main text, or Methods section.

n/a	Confirmed	
☐	☒	The exact sample size (*n*) for each experimental group/condition, given as a discrete number and unit of measurement
☐	☒	A statement on whether measurements were taken from distinct samples or whether the same sample was measured repeatedly
☐	☒	The statistical test(s) used AND whether they are one- or two-sided<br>*Only common tests should be described solely by name; describe more complex techniques in the Methods section.*
☐	☒	A description of all covariates tested
☐	☒	A description of any assumptions or corrections, such as tests of normality and adjustment for multiple comparisons
☐	☒	A full description of the statistical parameters including central tendency (e.g. means) or other basic estimates (e.g. regression coefficient) AND variation (e.g. standard deviation) or associated estimates of uncertainty (e.g. confidence intervals)
☐	☒	For null hypothesis testing, the test statistic (e.g. F, t, r) with confidence intervals, effect sizes, degrees of freedom and P value noted<br>*Give P values as exact values whenever suitable.*
☒	☐	For Bayesian analysis, information on the choice of priors and Markov chain Monte Carlo settings
☒	☐	For hierarchical and complex designs, identification of the appropriate level for tests and full reporting of outcomes
☒	☐	Estimates of effect sizes (e.g. Cohen's *d*, Pearson's *r*), indicating how they were calculated

Our web collection on statistics for biologists contains articles on many of the points above.

Software and code

Policy information about availability of computer code

Data collection	Matlab2017a; data https://github.com/kiigaya/Art
Data analysis	Matlab2018b, 2019b, 2021a, python, SPM12, code: https://github.com/kiigaya/Art

For manuscripts utilizing custom algorithms or software that are central to the research but not yet described in published literature, software must be made available to editors and reviewers. We strongly encourage code deposition in a community repository (e.g. GitHub). See the Nature Research guidelines for submitting code & software for further information.

Data

Policy information about availability of data

All manuscripts must include a data availability statement. This statement should provide the following information, where applicable:

- Accession codes, unique identifiers, or web links for publicly available datasets
- A list of figures that have associated raw data
- A description of any restrictions on data availability

The data that support the findings of this study are available from the corresponding author upon reasonable request. Also available here: https://github.com/kiigaya/Art

Field-specific reporting

Please select the one below that is the best fit for your research. If you are not sure, read the appropriate sections before making your selection.

☒ Life sciences ☐ Behavioural & social sciences ☐ Ecological, evolutionary & environmental sciences

For a reference copy of the document with all sections, see nature.com/documents/nr-reporting-summary-flat.pdf

Life sciences study design

All studies must disclose on these points even when the disclosure is negative.

Sample size	We analyzed each individual participant separately and treated six participants as six replication units.
Data exclusions	No exclusions.
Replication	We replicated previous behavioral results in a new sample across six participants
Randomization	N/A (no intervention, no group)
Blinding	N/A (no intervention, no group)

Reporting for specific materials, systems and methods

We require information from authors about some types of materials, experimental systems and methods used in many studies. Here, indicate whether each material, system or method listed is relevant to your study. If you are not sure if a list item applies to your research, read the appropriate section before selecting a response.

Materials & experimental systems

n/a	Involved in the study	
☒	☐	Antibodies
☒	☐	Eukaryotic cell lines
☒	☐	Palaeontology and archaeology
☒	☐	Animals and other organisms
☐	☒	Human research participants
☒	☐	Clinical data
☒	☐	Dual use research of concern

Methods

n/a	Involved in the study	
☒	☐	ChIP-seq
☒	☐	Flow cytometry
☐	☒	MRI-based neuroimaging

Human research participants

Policy information about studies involving human research participants

Population characteristics	Six volunteers (female: 6. age age 18-24 yr: 4; 25-34 yr: 1; 35-44 yr: 1. 4 White, 2 Asian) were recruited to our fMRI study. 1 participants completed master's degree or higher, 4 participants earned college degree as the highest degree, and 1 participant had a high-school degree as the highest degree. None of the participants possessed an art degree. All of the participants reported that they visit art museums less than once a month.
Recruitment	Through email list. This means that we invited participants who have email addresses and joined the mailing list. We also invited people who lived in southern California at the time of the study. This may create a potential bias.
Ethics oversight	Caltech IRB

Note that full information on the approval of the study protocol must also be provided in the manuscript.

Magnetic resonance imaging

Experimental design

Design type	Event-related task design.
Design specifications	On each trial, participants were presented with an image of the artwork on the computer screen for three seconds. Participants were then presented with a scale from 0, 1, 2, 3 in which they had to indicate how much they liked the artwork. The location of each numerical score was randomized across trials. Participants had to press a button of a button box that they hold with both hands to indicate their rating within three seconds, where each of four buttons

corresponded to a particular location on the screen from left to right. The left (right) two buttons were instructed to be pressed by their left (right) thumb. After a brief feedback period showing their chosen rating (0.5 sec), a center cross was shown for inter-trial intervals (jittered between 2 to 9 seconds). Each run consists of 50 trials. Participants were invited to the study over four days to complete twenty runs, where participants completed on average five runs on each day.

Behavioral performance measures: button press

Acquisition

Imaging type(s): Functional, Structual

Field strength: 3T

Sequence & imaging parameters: fMRI data were acquired on a Siemens Prisma 3T scanner at the Caltech Brain Imaging Center (Pasadena, CA). With a 32-channel radiofrequency coil, a multi-band echo-planar imaging (EPI) sequence was employed with the following parameters: 72 axial slices (whole-brain), A-P phase encoding, -30 degrees slice tilt with respect to AC-PC line, echo time (TE) of 30ms, multi-band acceleration of 4, repetition time (TR) of 1.12s, 54-degree flip angle, 2mm isotropic resolution, echo spacing of 0.56ms. 192mm x 192mm field of view, in-plane acceleration factor 2, multiband slice acceleration factor 4.

Positive and negative polarity EPI-based fieldmaps were collected before each run with very similar factors as the functional sequence described above (same acquisition box, number of slices, resolution, echo spacing, bandwidth and EPI factor), single band, TE of 50ms, TR of 5.13s, 90-degree flip angle.

T1-weighted and T2-weighted structural images were also acquired once for each participant with 0.9mm isotropic resolution. T1's parameters were: repetition time (TR) 2.4 s: echo time (TE), 0.00232 s; inversion time (TI) 0.8n s; flip angle, 10 degrees; , in-plane acceleration factor 2. T2's parameters were: TR 3.2 s; TE 0.564s; flip angle, 120 degrees; in-plane acceleration factor 2.

Area of acquisition: Whole brain

Diffusion MRI: ☐ Used ☒ Not used

Preprocessing

Preprocessing software: fMRI-prep 1.3.2

Normalization: Normalization was performed according to the default fMRI-prep procedure.

Normalization template: MNI

Noise and artifact removal: Motion regressors and aCompCor regressors were regressed out in the GLM analyses.

Volume censoring: fMRI-prep 1.3.2

Statistical modeling & inference

Model type and settings: Univariate analyses, first level only. Lasso regression.

Effect(s) tested: Liking ratings: t-tests; permutation test
Features: F-tests; permutation test

Specify type of analysis: ☐ Whole brain ☐ ROI-based ☒ Both

Anatomical location(s): Visual regions are taken from previous literature. Other regions are taken from the AAL atlas.

Statistic type for inference (See Eklund et al. 2016): cFWE p<0.05 with threshold at p<0.001 at the whole brain. Permutation test at p<0.001.

Correction: cFWE p<0.05 at the whole brain.

Models & analysis

n/a	Involved in the study
☐	☒ Functional and/or effective connectivity
☒	☐ Graph analysis
☐	☒ Multivariate modeling or predictive analysis

Functional and/or effective connectivity	Psychophysiological Interaction analysis
Multivariate modeling and predictive analysis	univariate regression analysis with cross-validation
